# LncRNAs: Novel Biomarkers for Pancreatic Cancer

**DOI:** 10.3390/biom11111665

**Published:** 2021-11-10

**Authors:** Soudeh Ghafouri-Fard, Mohadeseh Fathi, Tianyue Zhai, Mohammad Taheri, Peixin Dong

**Affiliations:** 1 Department of Medical Genetics, School of Medicine, Shahid Beheshti University of Medical Sciences, Tehran 1983535511, Iran; s.ghafourifard@sbmu.ac.ir; 2 Men’s Health and Reproductive Health Research Center, Shahid Beheshti University of Medical Sciences, Tehran 1983535511, Iran; mhds.fathi@gmail.com; 3 Department of Obstetrics and Gynecology, Hokkaido University School of Medicine, Hokkaido University, N15, W7, Kita-ku, Sapporo 0608638, Japan; zhaitianyue@gmail.com; 4 Skull Base Research Center, Loghman Hakim Hospital, Shahid Beheshti University of Medical Sciences, Tehran 1983535511, Iran

**Keywords:** lncRNAs, pancreatic cancer, biomarker, diagnosis, prognosis

## Abstract

Pancreatic cancer is one of the most deadly neoplasms and the seventh major cause of cancer-related deaths among both males and females. This cancer has a poor prognosis due to the lack of appropriate methods for early detection of cancer. Long non-coding RNAs (lncRNAs) have been recently found to influence the progression and initiation of pancreatic cancer. MACC1-AS1, LINC00976, LINC00462, LINC01559, HOXA-AS2, LINC00152, TP73-AS1, XIST, SNHG12, LUCAT1, and UCA1 are among the oncogenic lncRNAs in pancreatic cancer. On the other hand, LINC01111, LINC01963, DGCR5, MEG3, GAS5, and LINC00261 are among tumor suppressor lncRNAs in this tissue. In the current review, we summarize the roles of these two classes of lncRNAs in pancreatic cancer and discuss their potential as attractive diagnostic and prognostic biomarkers for pancreatic cancer. We also identified that the low expression of MEG3, LINC01963, and LINC00261 and the high expression of MACC1-AS1, LINC00462, LINC01559, and UCA1 were significantly correlated with worse survival in pancreatic cancer patients. Further research on these lncRNAs will provide new clues that could potentially improve the early diagnosis, prognostic prediction, and personalized treatments of patients with pancreatic cancer.

## 1. Introduction

Pancreatic cancer is one of the most deadly neoplasms as the whole number of deaths from this neoplasm is nearly equal to the number of affected individuals. This cancer is among the major causes of cancer-related demises among both males and females [1]. In numerous countries, the records of new affected individuals and deaths from pancreatic cancer have remained constant or slightly increased, most likely due to an increase in the occurrence of lifestyle-related risk factors (such as obesity, diabetes, and alcohol) or improvements in diagnostic procedures and cancer registry programs [2]. The most common subtype of this neoplasm is pancreatic ductal adenocarcinoma (PDAC) as the malignant cells originate from the ductal epithelium of the exocrine pancreas. PDAC is an aggressive tumor with poor response to treatment options [3]. Furthermore, the diagnosis of PDAC is delayed due to a lack of early diagnostic strategies. Another challenging issue in the field of PDAC therapy is the genetic and phenotypic heterogeneity of this type of malignancy [3]. These issues necessitate the identification of molecular pathways that are involved in the initiation and progression of pancreatic cancer. Long non-coding RNAs (lncRNAs) as a group of transcripts with regulatory functions have been found to partake in the pathogenesis of almost all types of neoplasms [4]. LncRNAs are RNAs longer than 200 nucleotides and have no protein-coding capacity. They are mainly transcribed by RNA polymerase II, yet other RNA polymerases are involved in their transcription [5]. While a number of lncRNAs are transcribed from intergenic regions (lincRNAs), others can overlap with other genes in sense or antisense directions [5]. The presence of a 7-methyl guanosine cap at the 5′ end, a polyadenylated tail at the 3′ end and splicing are all characteristics shared by lncRNAs and mRNAs. In addition, enhancer RNAs and promoter upstream transcripts are other types of lncRNAs that are transcribed from enhancer and promoter regions of genes, respectively [5]. LncRNAs have been found to induce numerous central phenotypes of cancer cells through interacting with other biomolecules such as DNA, proteins, and RNAs. Several cancer-related lncRNAs have been functionally annotated and have been proposed as potential cancer therapeutic targets [4]. In pancreatic cancer cells, numerous lncRNAs have been found to be dysregulated in association with progression of cancer. Several lncRNAs have been annotated as oncogenic or tumor suppressor lncRNAs in pancreatic cancer. In the current review, we focus on the summarization of the roles and the potential clinical implications of lncRNAs in pancreatic cancer. 

## 2. Oncogenic LncRNAs in Pancreatic Cancer

**Figure 1:**
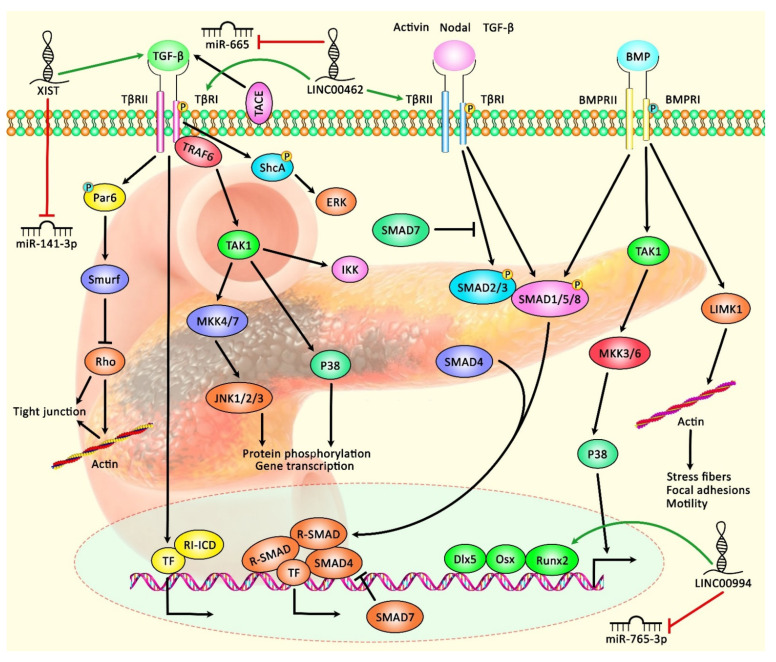
A schematic diagram shows the role of various lncRNAs in modulating the TGF-β/SMAD signaling pathway in pancreatic cancer. According to this cascade, when bioavailable TGF-β binds a homodimer of TβRII, transphosphorylation of the TβRI can trigger the activation of kinase activity. SMAD proteins, the substrates for TβRI kinases, are downstream of the BMP–analogous ligand–receptor systems. SMAD1, SMAD2, SMAD3, SMAD5, and SMAD8 can bind to membrane-bound serine/threonine receptors and are up-regulated via their kinase function. As a co-factor, the Co-SMAD (SMAD4) can bind to the up-regulated R-SMAD to create a complex that translocates into the nucleus. Consequently, I-SMAD (SMAD7) can deactivate the impacts of R-SMADs [36,37]. Previous studies have authenticated that several lncRNAs can play an effective role in regulating the TGF-β/SMAD cascade in pancreatic cancer. LINC00462 can up-regulate expression levels of TGFBR1 and TGFBR2 and activate the SMAD2/3 pathway in pancreatic cancer cells through down-regulating miR-665 expression [8]. Furthermore, lncRNA XIST can promote TGF-β2 expression via inhibiting the expression of miR-141-3p, thus enhancing cell proliferation, migration, and invasion of PC cells [33]. Green arrows indicate the up-regulation of target genes by lncRNAs; red arrows depict the inhibitory effects of lncRNAs.

Expression of lncRNAs has been appraised in pancreatic cancer tissues and cell lines using lncRNA microarray and qRT-PCR methods. These methods have resulted in the identification of numerous differentially expressed lncRNAs between neoplastic and non-neoplastic tissues. For instance, MACC1-AS1 has been identified as the most over-expressed lncRNA in pancreatic cancer tissues in a study conducted by Qi C et al. [6]. Expression of MACC1-AS1 was particularly elevated in patients who had poor survival. MACC1-AS1 silencing suppresses the proliferation and metastatic ability of pancreatic cancer cells. Mechanistically, MACC1-AS1 enhances the expression of PAX8 protein, which, in turn, increases aerobic glycolysis and activates NOTCH1 signaling. In addition, the expression of PAX8 is increased in pancreatic cancer tissues in correlation with levels of MACC1-AS1 and the prognosis of patients with pancreatic cancer. Thus, the MACC1-AS1/PAX8/NOTCH1 axis has been suggested as a putative target for the treatment of pancreatic cancer [6]. The association between expression levels of LINC00976 and pancreatic cancer progression has been assessed using in situ hybridization (ISH) and qRT-PCR methods. LINC00976 is up-regulated in pancreatic cancer tissues and cell lines in correlation with poor survival of patients. LINC00976 silencing inhibits proliferation, migratory potential and invasiveness of pancreatic cancer in vivo and in vitro. LINC00976 has been found to target ovarian tumor proteases OTUD7B. This protein deubiquitinates EGFR and influences the activity of MAPK signaling. Further studies have shown the role of the LINC00976/miR-137/OTUD7B axis in the modulation of the proliferation of pancreatic cancer cells [7]. Expression of LINC00462 in pancreatic cancer cells is induced by OSM. Up-regulation of this lncRNA has been accompanied by enhancement of cell proliferation, acceleration of cell cycle progression, and inhibition of cell apoptosis and adhesion. Moreover, LINC00462 up-regulation increases the migration and invasiveness of pancreatic cancer cells through enhancement of epithelial-mesenchymal transition (EMT) and accelerated growth and metastasis of pancreatic cancer in vivo. Moreover, over-expression of this lncRNA in clinical samples has been associated with larger tumor dimension, poor tumor differentiation, advanced TNM stage, and higher probability of metastasis in patients. Notably, LINC00462 has been demonstrated to have interaction with miR-665. Up-regulation of LINC00462 leads to the enhancement of expressions of TGFBR1 and TGFBR2 and the subsequent activation of the SMAD2/3 pathway in pancreatic cancer [8]. Table 1 shows the information on oncogenic lncRNAs in pancreatic cancer. Figure 1 illustrates the role of various lncRNAs in pancreatic cancer through regulating the TGF-β/SMAD signaling pathway.

## 3. Tumor Suppressor LncRNAs in Pancreatic Cancer

LINC01111 is a newly identified lncRNA that is significantly down-regulated in tissue and plasma samples gathered from patients with pancreatic cancer. This lncRNA has been found to exert tumor-suppressive effects. Notably, expression levels of LINC01111 have been inversely correlated with the TNM stage but positively correlated with the survival rate of patients with pancreatic cancer. LINC01111 can suppress the proliferation, cell cycle progression, invasiveness, and migratory potential of pancreatic cancer cells in vitro. Moreover, it can suppress the tumorigenic potential and metastatic ability of neoplastic cells in vivo. LINC01111 over-expression leads to the up-regulation of DUSP1 through sponging miR-3924. These events result in the inhibition of phosphorylation of SAPK, therefore inactivating SAPK/JNK signaling in pancreatic cancer cells [38]. LINC01963 is another down-regulated lncRNA in clinical samples of pancreatic cancer and cell lines. Over-expression of LINC01963 leads to suppression of colony formation, attenuation of cell cycle progression, and inhibition of proliferation and invasion of pancreatic cancer cells while enhancing the apoptosis rate in these cells. More importantly, short hairpin RNA targeting LINC01963 increases the tumorigenicity of pancreatic cancer cells in vivo. Functionally, LINC01963 decreases the expression of miR-641, a miRNA that down-regulates TMEFF2. Thus, LINC01963 suppresses the progression of pancreatic cancer through the miR-641/TMEFF2 axis [39]. MEG3 is another down-regulated lncRNA in pancreatic cancer cells, the expression of which has been inversely correlated with the expression of PI3K. In clinical samples, expression of MEG3 has been inversely correlated with tumor dimension, organ metastasis, and vascular invasion in pancreatic cancer. Functionally, MEG3 can suppress the progression of pancreatic cancer through regulation of the activity of PI3K/AKT/Bcl-2/Bax/cyclin D1/P53 and PI3K/AKT/MMP-2/MMP-9 axes [40]. On the other hand, the lncRNA GAS5 has been found to suppress metastasis of pancreatic cancer via the regulation of the miR-32-5p/PTEN axis [41].

**Figure 2:**
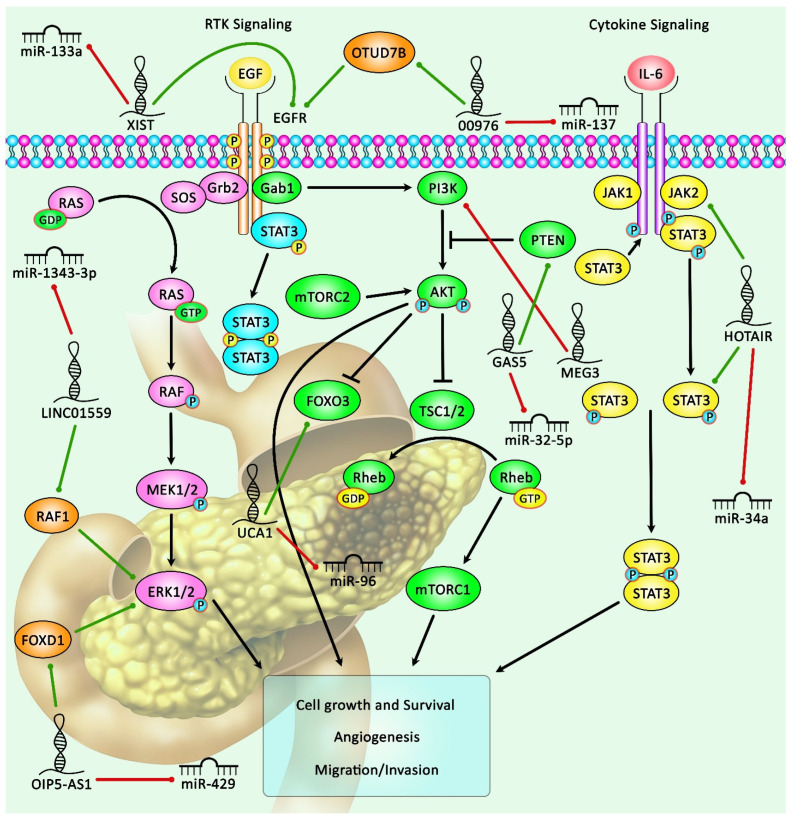
A schematic representation shows that several lncRNAs regulate the PI3K/AKT, MAPK/ERK and JAK2/STAT3 pathways in pancreatic cancer. Growth factor-driven RTK (e.g., EGFR) or cytokine (e.g., IL- 6) signaling can trigger the activation of PI3K/AKT, MAPK/ERK, and JAK2/STAT3 cascades. LncRNAs can affect the activity of these cascades. For instance, HOTAIR can trigger the activation of the JAK2/STAT3 pathway via down-regulating miR-34a expression, thus promoting invasion and migration of pancreatic ductal adenocarcinoma [12]. In addition, GAS5 can up-regulate PTEN expression by down-regulating the expression level of miR-32-5p, therefore inhibiting pancreatic cancer metastasis [41]. LINC01559, through sponging miR-1343-3p, can up-regulate RAF1 expression that can further activate the ERK signaling pathway, thereby enhancing pancreatic cancer progression and metastasis [15]. Green arrows indicate the up-regulation of target genes modulated via lncRNAs; red arrows depict the inhibitory effects.

Several tumor suppressor lncRNAs exert prominent effects on cell apoptosis. For instance, DGCR5 functions as a molecular sponge for miR-27a-3p, a miRNA that regulates the expression of BNIP3. Forced up-regulation of DGCR5 in pancreatic cancer cells leads to the down-regulation of miR-27a-3p. Furthermore, DGCR5 regulates the expression of BNIP3 and the activity of p38 MAPK via sponging miR-27a-3p. The miR-27a-3p/BNIP3 axis has been found to be the main mediator of the pro-apoptotic effects of DGCR5 [42]. Table 2 shows the list of down-regulated lncRNAs in pancreatic cancer. Figure 2 shows the role of several lncRNAs in regulating the PI3K/AKT, MAPK/ERK, and JAK2/STAT3 cascades in pancreatic cancer.

## 4. Diagnostic Role of LncRNAs in Pancreatic Cancer

The diagnostic role of lncRNAs in pancreatic cancer is appraised by depicting receiver operating characteristic (ROC) curves. The calculated values for the area under these curves (AUC values) for a number of these lncRNAs are more than 0.8, suggesting the potential of these lncRNAs as diagnostic markers for pancreatic cancer (Table 3). For instance, the expression of LINC00675 is firstly assessed in a small cohort of PDAC tissues and chronic pancreatitis tissues through microarray screening. At the next step, these results are validated in larger cohorts of patients using the qRT-PCR method. Over-expression of LINC00675 is significantly correlated with lymph node metastasis, perineural invasion, and poor clinical outcome of patients with pancreatic cancer. Notably, this lncRNA has a 0.893 AUC value for predicting the progression of pancreatic cancer within one year. Moreover, the AUC value for the prediction of tumor progression within six months is 0.928. Thus, LINC00675 is a potential diagnostic marker for the prediction of recurrence in PDAC patients following radical surgical resection [45]. 

C9orf139 is another up-regulated lncRNA in the tissues and sera of patients with pancreatic cancer that has diagnostic value in clinical settings since the AUC value of this lncRNA has been estimated to be 0.923. Over-expression of this lncRNA has been associated with a higher possibility of cancer progression to advanced stages, lymph node metastasis, and poor differentiation [46]. 

Moreover, serum levels of UFC1 expression are relatively higher in patients with pancreatic cancer compared with healthy controls. ROC curve analyses have shown that the serum levels of this lncRNA can separate pancreatic cancer patients from healthy subjects with an AUC value of 0.810. Moreover, serum levels of UFC1 have been associated with lymph nodes involvement, metastases to distant organs, and clinical stage [47]. 

## 5. Prognostic Role of LncRNAs in Pancreatic Cancer

**Figure 3:**
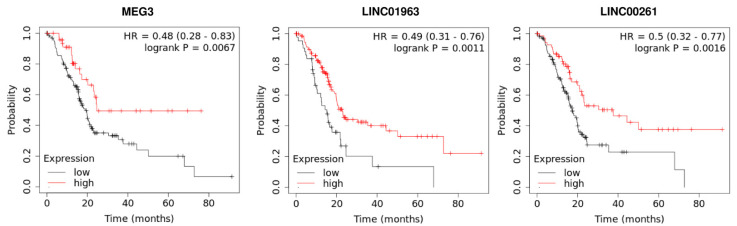
The prognostic value of lncRNA MEG3, LINC01963, and LINC00261 in pancreatic cancer patients was analyzed using the KM-plotter database.

**Figure 4:**
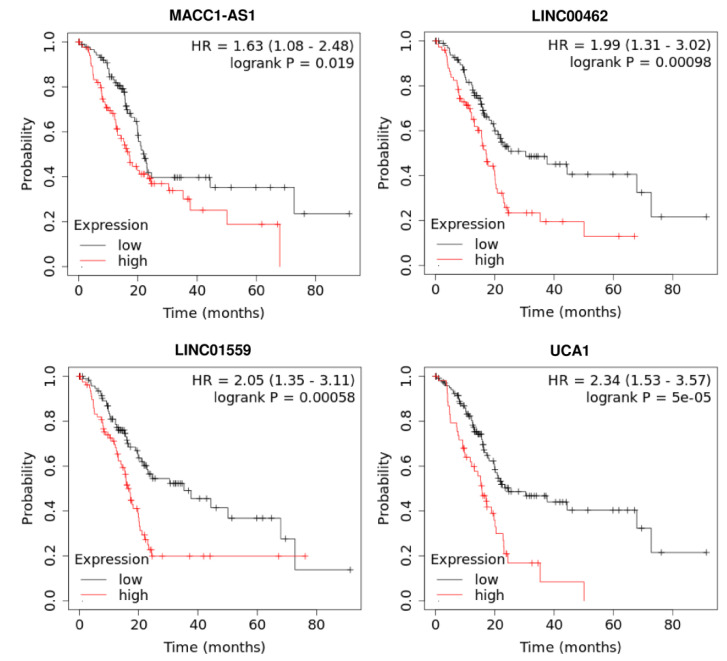
The prognostic value of lncRNA MACC1-AS1, LINC00462, LINC01559, and UCA1 in pancreatic cancer patients was investigated using the KM-plotter database.

The prognostic role of lncRNAs in pancreatic cancer has been validated in several investigations (Table 4). Many lncRNAs with potential application as diagnostic markers have also been demonstrated to have prognostic potential. For instance, UFC1 not only serves as a diagnostic marker but also facilitates the prediction of the prognosis of pancreatic cancer. Based on the results of the Kaplan-Meier analysis, over-expression of UFC1 has been associated with shorter progression-free survival and overall survival rates. Multivariate analyses have also shown the potential of UFC1 expression levels as an independent prognostic factor for pancreatic cancer [47]. UNX1-IT1 is another lncRNA whose expression levels have been correlated with differentiation grade of tumors, lymph node involvement, and clinical stage. Over-expression of RUNX1-IT1 has also been correlated with a significant reduction in overall survival. Based on univariate and multivariate Cox regression analyses, over-expression of RUNX1-IT1 has been identified as a factor that increases the risk of mortality from pancreatic cancer [52]. ENSG00000254041.1 is another novel lncRNA whose expression is particularly elevated in pancreatic cancer samples with high EMT signature scores. Multivariate analyses have proposed ENSG00000254041.1 as an independent factor for pancreatic cancer [53]. Previous survival analyses performed on pancreatic cancer patients revealed that patients with low MEG3 expression had a worse prognosis [54]. Consistent with this result, our Kaplan–Meier analysis by the KM-plotter database has shown that MEG3 expression is positively correlated with longer overall survival in pancreatic cancer patients (Figure 3). Our survival analysis also indicated that an increased expression of lncRNA LINC01963 and LINC00261 were significantly associated with better overall survival in pancreatic cancer patients (Figure 3). In contrast, upregulation of lncRNA MACC1-AS1, LINC00462, LINC01559, and UCA1 predicted shorter overall survival in pancreatic cancer patients (Figure 4). 

## 6. Discussion

Non-coding RNAs have important roles in the pathoetiology of human disorders [59]. LncRNAs can be classified based on their genomic locations into four classes, namely, intergenic lncRNAs (such as LINC00462 and LINC00958), which are transcribed from intergenic regions of either sense or antisense strands; intronic lncRNAs, which are transcribed totally from the intronic regions of protein-coding genes; sense lncRNAs, which are transcribed from the sense strand of protein-coding genes and encompass exons; and antisense lncRNAs (such as MACC1-AS1 and HOXA-AS2), which are transcribed from the antisense strand of protein-coding genes [60]. These transcripts have a crucial impact on gene expression through different mechanisms, namely, transcriptional interference, chromatin remodeling, regulation of splicing events, modulation of translation through binding to translation factors or ribosomes, acting as competing endogenous RNAs for miRNAs, altering localization of proteins, modulation of telomere replication, and RNA interference [60,61]. Based on these diverse roles in cellular and biochemical processes, lncRNAs have been suggested as disease biomarkers and therapeutic targets. Suppression of expression of over-expressed lncRNAs has been suggested as a therapeutic option for human diseases. This aim has been accomplished through the application of RNAi methods, degradation of lncRNAs by RNase H, antisense oligonucleotides, or application of the genome-editing strategy CRISPR/Cas9 [62]. 

LncRNAs can affect the growth, migration, and invasion of pancreatic cancer [63]. These transcripts have been suggested as a key class of pervasive genes participating in tumorigenesis and metastasis [64]. They have been demonstrated to partake in the pathobiology of pancreatic cancer via different mechanisms, among which is the modulation of cancer-related pathways such as JAK2/STAT3, EGFR/MAPK, ERK, NOTCH, and PTEN pathways. The EMT process is an important process for the progression of cancer metastasis and is affected by several lncRNAs in pancreatic cancer. LINC00462, LINC00958, SNHG12, and OIP5-AS1 are among lncRNAs whose roles in the progression of EMT have been validated in pancreatic cancer. 

Although several mechanisms have been found as routes of participation of lncRNAs in the carcinogenesis of pancreatic cancer at the molecular level, the interaction between lncRNAs and miRNAs seems to be the most appreciated route. LINC00976/miR-137, LINC00462/miR-665, LINC00976/miR-137, LINC01559/miR-1343-3p, LINC00152/miR-150, TP73/miR-141, XIST/miR-141-3p, SNHG12/miR-320b, LUCAT1/miR-539, LINC00958/miR-330-5p, CRNDE/miR-384, SNHG7/miR-146b-5p, ZEB2-AS1/miR-204, PVT1/miR-519d-3p, SNHG14/miR-613, SBF2-AS1/miR-122-5p, LINC00994/miR-765-3p, and LINC01207/miR-143-5p are among the identified pairs between oncogenic lncRNAs and tumor suppressor miRNAs that are sponged by these lncRNAs. On the other hand, some tumor suppressor lncRNAs have been found to act as sponges for oncogenic miRNAs. LINC01111/miR-3924, LINC01963/miR-641, DGCR5/miR-27a-3p, GAS5/miR-32-5p, and LINC00261/miR-23a-3p axes are examples of the latter type of lncRNA/miRNA interactions in the context of pancreatic cancer. LncRNAs might also act as decoys, scaffolds, and enhancers [65]. However, these modes of action have not been completely assessed in the context of pancreatic cancer. The main challenge in the field of lncRNAs is that the underlying mechanism of action of many of these transcripts is not yet entirely clear. Additional perception of the biological impact and function of lncRNAs would require further investigations to be accomplished, which might result in the discovery of some currently unidentified modes of action. Another perplexing factor in comprehending the mechanism of lncRNA function is that lncRNAs might use more than one mode of action to exert their regulatory roles on gene transcription [66]. 

Several lncRNAs, including lncRNA-UFC1, RP11-263F15.1, ABHD11-AS1, LINC00675, HULC, and C9orf139, have been shown to have the potential to be included in diagnostic approaches for pancreatic cancer. Therefore, combinations of expression amounts of these lncRNAs might enhance the efficiency of diagnosis of pancreatic cancer, particularly using non-invasive methods conducted on biofluids obtained from patients. 

Expression of lncRNAs can also predict the behavior of pancreatic cancer. RUNX1-IT1, ENSG00000254041.1, MALAT1, LOC285194, LncRNA-UFC1, RP11-263F15.1, BC008363, MEG3, and HULC are among lncRNAs whose expressions have been correlated with the survival of patients with pancreatic cancer. 

Oncogenic lncRNAs can be targeted by strategies such as shRNAs or antisense oligonucleotides [67]. Another promising strategy in this regard is the use of emerging routes of genome editing such as the CRISPR system [68]. However, several biosafety and bioavailability issues should be solved before the wide application of these techniques in clinical settings. Moreover, the context-dependent functions of lncRNAs should be completely clarified to avoid any detrimental effects when applying anti-lncRNA therapeutic modalities. 

In brief, lncRNAs have pivotal roles in the pathogenesis of pancreatic cancer and have applications in diagnostic and prognostic approaches to this cancer. Based on the results of in vitro and in vivo experiments, modulation of expression of lncRNAs can be regarded as an appropriate strategy for the treatment of pancreatic cancer. The lncRNAs that have been previously reported in the literature and those lncRNAs identified in this study have the potential to serve as novel diagnostic, prognostic, and individualized treatment-predictive biomarkers for pancreatic cancer. 

## Figures and Tables

**Table 1:** List of up-regulated lncRNAs in pancreatic cancer (ANT: adjacent non-cancerous tissue; cell lines were used for functional studies, apoptotic assays, and identification of partners of lncRNAs).

LncRNA	Sample	Cell Line	Interaction	Signaling Pathway	Clinical Properties	Method	Function	Ref.
**C9orf139**	54 pairs of tumor and ANTs	AsPC-1, BxPC3, PANC1, PaCa-2, SW-1990, HPDE6-C7	miR-663a/Sox12	_	Tumor stage, lymph nodemetastasis	qRT-PCR, Western blotting, RNA immunoprecipitation, RNA pull-down, luciferase reporter assay	High expression of LncRNA C9orf139 is associated with the poor clinicopathological feature of PC patients	[9]
**CRNDE**	58 pairs of tumor and ANTs	SW-1990, PANC-1,CAPAN-1, JF305 BxPC-3,HPDE6-C7	miR-384	_	Tumordifferentiation, tumor size, TNM stage, and lymph nodal metastasis	qRT-PCR, luciferase reporter assays, Western blotting, immunohistochemistry (IHC) analysis	LncRNA CRNDE plays an oncogenic role in PC tissue and cell lines via directly targeting miR-384	[10]
**H19**	139 invasive ductal carcinoma samples	PANC-1		-	-	In situ hybridization, DNA microarray analysis, qRT-PCR	H19 affects cell motility but not cell growth rate	[11]
**HOTAIR**	_	HPDE6-C7, SU.86.86, CFPPAC-1, SW-1990, PL45	miR-34a	JAK2/STAT3 Pathway	_	qRT-PCR, Western blotting, RNA pull-down	LncRNA HOTAIR can activate the JAK2/STAT3 pathway by targeting miR-34 and then enhancing the proliferation and invasion of PC cells	[12]
**HOTTIP**		Panc-1, L3.6pL, and MiaPaCa2	HOXA10, HOXB2, HOXA11, HOXA9, and HOXA1	-	-	Illumina Human V.3 HT12 Beadchip array	HOTTIP regulates the proliferation, apoptosis, and migration of PC cells	[13]
**HOXA-AS2**	16 pairs of tumor and ANTs, 12 pairs of tumor and ANTs	AsPC-1, BxPC-3, PANC-1	enhancer of zeste homolog 2 (EZH2), lysine-specific demethylase 1 (LSD1)	_	_	qRT-PCR	lncRNA HOXA-AS2 plays an oncogenic role in pancreatic cancer tissue	[14]
**L** **INC** **00976**	_	CFPAC-1, MIA-PaCa-2, PANC-1, BxPC-3,CFPAC-1, ASPC-1, Panc03.27, Capan-2	miR-137/ OTUD7B	EGFR/MAPK signaling pathway	Tumor size, lymph node metastasis, perineural invasion, vascularinvasion, distant metastasis ability	In situ hybridization (ISH), qRT-PCR	LINC00976 plays an oncogenic role in pancreatic cancer tissue and promotes invasion, migration, and proliferation via up-regulating OTUD7B and then targeting miR-137	[7]
**LINC00462**	_	SW-1990, BxPC3,PANC-1, AsPC-1, CFPAC-1, HPDE6-C7	miR-665, TGFBR1, TGFBR2	SMAD2/3 signaling pathway	Large tumor size, poor tumor differentiation, TNM stage, distant metastasis	qRT-PCR, CCK-8 assay, Western blotting, flow cytometry analyses, immunofluorescence	Over-expression of LINC00462 significantly promotes EMT and cell proliferation and suppresses cell apoptosis via up-regulating TGFBR1 and TGFBR2	[8]
**LINC01559**	55 pairs of tumor and ANTs	AsPC-1, BxPC-3, PANC-1, MIA- PaCa-2, SW-1990, HPDE	miR-1343-3p/RAF1	ERK signaling pathway	Large tumors, lymph node metastasis,	RT-qPCR, RIP assay, CCK-8 assay, Western blotting, immunohistochemistry (IHC)	High expression of LINC01559 enhances proliferation of pancreatic cancer cells and metastasis by up-regulating Raf1 and activating the ERK pathway	[15]
**LINC00152**	28 pairs of tumor and ANTs	BxPC3, Panc1, AsPC1, SW-1990, HPDE6-C7	miR-150	_	_	qRT-PCR, CCK-8 assay, EDU assay, luciferase reporter assay	LINC00152 can suppress miR-150 and then promote pancreatic cancer cells progressions	[16]
**LINC00958**	_	PANC-1, Capan-2, SW-1990, BxPC-3,HPDE	miR-330-5p	_	_	qRT-PCR, Western blotting, fluorescent in situ hybridization (FISH), RNA immunoprecipitation (RIP)	LINC00958 enhances the EMT process and metastatic ability of PC cells	[17]
**LUCAT1**	60 pairs of tumor and ANTs	BxPC-3, Capan2, AsPC-1, PANC-1, HPDE6c7	miR-539	_	tumor size, lymphatic invasion.	qRT-PCR, in situ hybridization, Western blotting	LUCAT1 can enhance the invasion ability of cells by targeting miR-539	[18]
**LINC00994**	10 pairs of tumor and ANTs	PANC-1, AsPC-1, SW-1990	miR-765-3p/RUNX2	_	_	Microarrays, qRT-PCR, flow cytometry, luciferase assay, Western blotting	LINC00994 acts as an oncogene and its inhibition can suppress RUNX2 by targeting miR-765-3p	[19]
**LINC01207**	36 pairs of tumor and ANTs	PANC-1, BxPC-3, Mpanc-96, PaTu-8988	miR-143-5p	_	_	qRT-PCR, RNA pull-down, RNA immunoprecipitation (RIP), flow cytometry, immunofluorescence staining, Western blotting	Its inhibition can induce apoptosis and autophagy activity of PC cells via targeting miR-143-5p	[20]
**MACC1-AS1**	98 pairs of tumor and ANTs, 124 pairs of tumor and ANTs	BxPC-3, PANC-1, MIA-PaCa-2, KP-2, AsPC-1, Capan-1	PAX8	NOTCH1 signaling pathway	_	lncRNA microarray, qRT-PCR, luciferase analyses, RNAimmunoprecipitation	High expression of LncRNA MACC1-AS1 can induce pancreatic cancer cells proliferation and promote metastasis through regulating the PAX8/NOTCH1 signaling pathway	[6]
**OIP5-AS1**	110 pairs of tumor and ANTs	PANC-1, BxPC-3, AsPC-1, CFPAC-1, HPDE6-C7	miR-429, FOXD1, ERK pathway	ERK pathway	Tumor size, distant metastasis, TNM stage	qRT-PCR, RNA immunoprecipitation, RNA pull-down, luciferase reporter assay, Western blotting	High expression of LncRNA OIP5-AS1 can increase EMT process, invasion, and PC cell proliferation via activating the ERK pathway	[21]
**PVT1**	30 pairs of tumor and ANTs	HPAC, DANG, BxPC-3, PANC1, ASPC-1, H6C7	miR-519d-3p	glycolysispathway	lymph node metastasis	qRT-PCR, Western blotting, RNA immunoprecipitation (RIP) assay, RNA pull-down assay, immunohistochemistry (IHC)	PTV1 induces downregulation of miR-519d-3p and then promotes the progression of pancreatic cancer	[22]
**RP11-567G11.1**	78 tumor tissues and 7 non-tumor tissues	SW-1990, BxPC-3, PANC-1	_	NOTCH signaling pathway	_	In situ hybridization, CCK8 and flow cytometry, Western blotting, qPCR	Inhibition of LncRNA RP11-567G11.1 can induce apoptosis and suppress cancer cell proliferation	[23]
**SBF2-AS1**	_	PANC-1, BxPC-3, SW-1990, Capan2, THP-1	miR-122-5p	SMAD signaling pathway	_	Flow cytometry, RNA-fluorescence in situ hybridization(FISH), qRT-PCR, Western blotting, RNA immunoprecipitation, RNA pull-down assays	The expression level of SBF2-AS1 is increased in M2 macrophage exosomes and plays an oncogenic role in pancreatic cancer tissue	[24]
**SNHG7**	50 pairs of tumor and ANTs	PANC-1, SW-1990, BxPC-3 AsPC-1, HPDE	miR-146b-5, roundabout homolog 1(Robo1)	_	Tumor size, distant metastasis, lymph node metastasis,	qRT-PCR, Flow cytometry analysis, luciferase reporter assay, RNA immunoprecipitation (RIP) assay, RNA pull-down assay, Western blotting	High expression of LncRNA SNHG7 can promote the progression of PC by positively affecting Robo1	[25]
**SNHG12**	15 pairs of tumor and ANTs	HPDE6, BxPC-3, CAPAN1, PANC1, SW-1990	miR-320b	_	_	qRT-PCR, flow cytometry, luciferase assay	LncRNA SNHG12 can increase the invasion, EMT, and proliferation of cancer cells by negatively affecting miR-320b	[26]
**SNHG14**	45 pairs of tumor and ANTs	CFPAC-1, BxPC-3, L3.6pl Panc-1, HPDE6C7	miR-613	_	Poor tumor differentiation, advanced TNM stage, nodal metastasis	qRT-PCR, fluorescent in situ hybridization, flow cytometry, Western blotting	Increased expression of SNHG14 can promote the progression of pancreatic cancer by inhibiting caspase-3 activity and down-regulation of miR-613	[27]
**SNHG15**	48 pairs of tumor and ANTs	AsPC-1, BxPC-3, HPDE6	zeste homolog 2	_	tumor size, TNM stage,lymph node, metastasis	qRT-PCR, Flow cytometry, Western blotting, RNA immunoprecipitation, chromatin immunoprecipitation (ChIP)	SNHG15 plays an oncogenic role in pancreatic cancer tissue by inversely regulating target genes	[28]
**SPRY4-IT1**	_	BxPC-3, PANC-1	Cdc20	_	_	qRT-PCR, Western blotting, wound healing assay, Transwell assay	SPRY4-IT1 acts as an oncogene in PC tissue, and its inhibition induces depletion of PC progression	[29]
**TP73** **-AS1**	77 pairs of tumor and ANTs	HPDE6-C7, SW-1990, CAPAN-1, JF305, PANC-1, BxPC-3	miR-141	_	TNM stage, lymph node metastasis	qRT-PCR, luciferase reporter assays, Western blotting	High expression of lncRNA TP73-AS1 induces migration, invasion, and PC cell proliferation	[30]
**UCA1**	120 pairs of tumor and ANTs	PANC-1, BxPC-3, Capan-1, SW-1990, HPDE6C-7	_	_	Tumor size, depth of invasion, CA19-9 level, tumor stage	qRT-PCR, flow cytometry, Western blotting,	Low expression of LncRNA UCA1 can reduce the proliferation of PC cells and induce cell cycle arrest	[31]
**UCA1**	36 pairs of tumor and ANTs	HPC-Y5, PANC-1, SW-1990, AsPC-1	miR-96/FOXO3	_	_	qRT-PCR, Western blotting, immunohistochemistry, flow cytometry, luciferase assay, RNA in situ hybridization	LncRNA UCA1 acts as an oncogene in PC tissue and cell lines via negative regulating miR-96	[32]
**XIST**	30 pairs of tumor and ANTs	PANC-1, HEK293T	miR-141-3p, TGF-β2	TGF-β signaling pathway	_	qRT-PCR, luciferase reporter assay, Western blotting	LncRNA XIST plays an oncogenic role in PC tissue through targeting miR-141-3p and the TGF-β signaling pathway	[33]
**XIST**	64 pairs of tumor and ANTs	H6c7, Patu8988,SW-1990, BxPC-3, AsPC-1, CFPAC-1, PANC-1	miR-133a/EGFR	EGFR/Akt signaling	Larger tumor size, perineuralinvasion, lymph node metastasis, shorter overall survival	qRT-PCR, BrdU cell proliferation assay, luciferase reporter assay	LncRNA XIST can induce PC cell proliferation through negatively regulating miR133a and positively regulating EGFR	[34]
**ZEB2-AS1**	39 pairs of tumor and ANTs	AsPC-1, HPAC, Cfpac-1, PANC-1, HPDE	miR-204/ HMGB1	_	_	q-RT-PCR, Western blotting immunofluorescence assay, luciferase reporter assay, RNA-binding protein immunoprecipitation (RIP) assay, LncRNA array	Overexpression of LncRNA ZEB2-AS1 induces cell proliferation and invasion by negatively affecting miR-204	[35]

**Table 2:** List of down-regulated lncRNAs in pancreatic cancer (ANTs: adjacent non-cancerous tissue).

LncRNA	Sample	Cell Line	Interaction	Signaling Pathway	Clinical Properties	Method	Function	Ref.
**ENST00000480739**	35 patients with pancreatic cancer	ASPC-1, BXPC-3, CFPAC-1, PANC-1 and SW1990	OS-9	-	Tumor node metastasis stage and lymph node metastasis	Transwell invasion assay, ELISA, Western blot	ENST00000480739 participates in tumor metastasis and progression	[43]
**LINC01111**	_	HPDE, PANC-1, MIA-PaCa-2,SW-1990, Capan-2, Panc 03.27, BxPC-3, CFPAC-1	miR-3924	SAPK/JNK signaling pathway	TNM stage (negatively), survival (positively)	qRT-PCR, EdU incorporation assay, scratch wound healing assays, Western blotting, RNA microarrays, in situ hybridization	LINC01111 plays a tumor-suppressive role in PC tissue and cell lines via inhibition of the SAPK/JNK signaling pathway	[38]
**LINC01963**	67 pairs of tumor and ANTs	PANC-1, CFPAC-1, BxPC-3, SW-1990, AsPC1, HPDE6-C7	miR-641/TMEFF2	_	Distantmetastasis, TNM stag	qRT-PCR, flow cytometry assay, luciferase assay, RNA immunoprecipitation, Western blotting	High expression of LncRNA LINC01963 can induce inhibition of pancreatic cancer progression via negatively regulating miR-641	[39]
**DGCR5**	20 pairs of tumor and ANTs	SW-1990, PANC-1, HPDE6-C7	miR-27a-3p/BNIP3	p38 MAPK pathway	_	qRT-PCR, Western blotting, RNA immunoprecipitation (RIP), RNA pull-down assay, luciferase reporter assay, flow cytometric (FCM) analysis	Down-regulation of lncRNA DGCR 5 affects apoptosis through regulating BNIP3 and the p38 MAPK pathway	[42]
**MEG3**	30 pairs of tumor and ANTs	PANC-1	PI3K protein	PI3K/AKT/Bcl-2/Bax/cyclin D1/P53 and PI3K/AKT/MMP-2/MMP-9 signaling pathways	Tumor size, metastasis, and vascular invasion	Immunohistochemistry (IHC) assay, qRT-PCR, Western blotting	LncRNA MEG 3 acts as a tumor-suppressor in PC tissue and cell lines	[40]
**GAS5**	22 pairs of tumor and ANTs	PANC-1, BxPC-3, HPDE6-C7	miR-32-5p	PTEN signaling pathway	_	qRT-PCR, Western blotting, flow cytometry analysis, RNA immunoprecipitation (RIP) assay, RNA pull-down assay	GAS5 exhibits tumor suppressor activity in PDAC tissue samples	[41]
**LINC00261**	_	CFPAC-1, BxPC-3, PANC-1, AsPC-1, HPDE6-C7	miR-23a-3p	_	_	qRT-PCR, flow cytometry, Western blot	A low expression level of LINC00261 can promote PC progression by targeting miR-23a-3p	[44]

**Table 3:** Diagnostic role of lncRNAs in pancreatic cancer (ANT: adjacent non-cancerous tissue).

LncRNA	Expression Pattern	Detection Method for LncRNAs	Sample	Area Under the Curve (AUC)	References
LncRNA-UFC1	Up-regulation	qRT-PCR	48 serum samples of patients	0.810	[47]
RP11-263F15.1	Up-regulation	Microarray, qRT-PCR	71 pairs of tumor and ANTs	0.843	[48]
ABHD11-AS1	Up-regulation	qRT-PCR	15 serum samples of patients and 30 healthy individuals	0.887	[49]
LINC00675	Up-regulation	Microarray, qRT-PCR	45 pairs of tumor and ANTs	0.928	[45]
HULC	Up-regulation	qRT-PCR	60 serum samples of patients and 60 healthy individuals	0.856	[50]
C9orf139	Up-regulation	qRT-PCR	54 pairs of tumor and ANTs	0.923	[46]
PVT1	Up-regulation	qRT-PCR	Salivary samples from 55 patients with resectable pancreatic cancer, 20 patients with benign pancreatic lesions, and 55 normal controls	0.84 (cancer vs. benign lesion), 0.90 (cancer vs. healthy state)	[51]
HOTAIR	Up-regulation	qRT-PCR		0.86 (cancer vs. benign lesion), 0.88 (cancer vs. healthy state)	

**Table 4:** Prognostic role of lncRNAs in pancreatic cancer (ANT: adjacent non-cancerous tissue).

LncRNA	Expression Pattern	Sample	Kaplan–Meier Analysis	Multivariate Analysis	References
**RUNX1-IT1**	Up-regulated	83 tumor tissues and 38 ANTs and 15 normal pancreatic tissues	Overexpression of lncRNA RUNX1-IT1 was associated with poor overall survival	Expression of lncRNA RUNX1-IT1 was identified as an independent prognostic factor for pancreatic cancer patients	[52]
**ENSG00000254041.1**	Up-regulated	70 pairs of tumor and ANTs	Its high expression was associated with poor overall survival	Expression of lncRNA ENSG00000254041.1 can be an independent predictor of pancreatic cancer survival	[53]
**MALAT1**	Up-regulated	45 pairs of tumor and ANTs	Its high expression was associated with poor disease-free survival	Expression of lncRNA MALAT1 can be an independent prognostic factor for disease-specific survival in patients	[54]
**LOC285194**	Down-regulated	85 pairs of tumor and ANTs	Low expression of lncRNA LOC285194 was associated with poor overall survival	Low expression of lncRNA LOC285194 can be an independent poorprognostic factor in pancreatic cancer patients	[55]
**LncRNA-UFC1**	Up-regulated	48 serum samples of patients	Overexpression of lncRNA-UFC1 was associated with shorter progression-free survival and overall survival	Expression levels of lncRNA-UFC1 were identified as independent prognostic factors in patients	[47]
**RP11-263F15.1**	Up-regulated	71 pairs of tumor and ANTs	Increased lncRNA RP11-263F15.1 expression level was associated with poor overall survival	The expression level of lncRNA RP11-263F15.1 was not independent of prognostic factors in patients	[48]
**BC008363**	Down-regulated	30 pairs of tumor and ANTs	Overexpression of lncRNA BC008363 indicated better overall survival	_	[56]
**MEG3**	Down-regulated	25 pairs of tumor and ANTs	Increased LncRNA MEG3 expression was associated with longer overall survival	_	[57]
**HULC**	Up-regulated	25 pairs of tumor and ANTs	A high expression level of LncRNA HULC was associated with shorter overall survival	The expression level of LncRNA HULC identified as an independentpredictor for overall survival	[58]
